# On the emergence of the in–out effect across trials: two items do the trick

**DOI:** 10.1007/s00426-022-01715-6

**Published:** 2022-07-22

**Authors:** Sascha Topolinski, Lea Boecker, Charlotte S. Löffler, Beatriz Gusmão, Moritz Ingendahl

**Affiliations:** 1 Department of Psychology, University of Cologne, Richard-Strauss-Straße 2, 50931 Cologne, Germany; 2 Department of Economic Psychology, Social Psychology and Experimental Methods, University of Lüneburg, Lüneburg, Germany; 3 Department of Psychology, University of Mannheim, Mannheim, Germany

## Abstract

Individuals prefer letter strings whose consonantal articulation spots move from the front of the mouth to the back (e.g., BAKA, inward) over those with a reversed consonant order (e.g., KABA, outward), the so-called in–out effect. The present research explores whether individuals hold an internal standard or scheme of consonant order that triggers this effect. If this were the case, the in–out effect should already occur in one-trial between-subjects designs. If not, the in–out effect should emerge over the course of trials in within-subjects designs. In Experiments 1a–e (1b–e preregistered; total *N* = 2973; German, English, and Portuguese samples) employing a one-trial between-subjects design, no in–out effect was found. In Experiment 2 (*N* = 253), employing within-subjects designs with either 1, 5, 10, 30, or 50 trials per consonant order category (inward vs. outward), the in–out effect was absent in the first trial, but already surfaced for the first 2 trials, reached significance within the first 10 trials and a solid plateau within the first 20 trials. Of the four theoretical explanations, the present evidence favors the fluency/frequency and letter-position accounts and is at odds with the eating-related embodiment and easy-first accounts.

## On the emergence of the in–out effect across trials: two items do the trick

Consonants are produced at different spots in the mouth, varying from the front of the mouth to the back (Titze, 1994). People prefer letter strings in which the articulation spots of the consonants move from the front of the mouth to the back (e.g., PAK, first lips then back tongue; inward) over letter strings in which the consonantal articulation spots move from the back of the mouth to the front (e.g., KAP, first back tongue then lips; outward). This so-called in–out effect (Topolinski et al., 2014) biases a wide range of judgments and even behavior, such as basic word liking (Topolinski et al., 2014), memory for words (Lindau & Topolinski, 2018b), attitudes towards persons bearing inward or outward strings as names (Garrido et al., 2019; Silva & Topolinski, 2018), consumer attitudes towards products bearing in/out strings as brands (Godinho & Garrido, ; Ingendahl & Vogel, 2022a; Pathak et al., 2021; Rossi et al., 2017; Topolinski, 2017a, 2017b; Topolinski & Boecker, 2016b; Topolinski et al., 2015), and even food consumption (Rossi et al., 2017).

Thus far, the in–out effect has been documented in German, English, French, Portuguese, Ukrainian, Turkish, and Japanese native speakers (Godinho & Garrido, 2016; Godinho et al., 2019a, 2019b; Körner & Rummer, 2021; Motoki & Pathak, 2022; Rossi et al., 2017; Topolinski et al., 2014) and occurs for even highly complicated string structures (e.g., FOLOKOLOF vs. KOLOFOLOK, Topolinski & Bakhtiari, 2016) and under sub-optimal presentation conditions, such as very brief exposure (Gerten & Topolinski, 2020), aural presentation (Topolinski & Boecker, 2016a), and even concurrent oral interference (Lindau & Topolinski, 2018a).

Its driving mechanisms are hotly debated (for a succinct review, see Ingendahl et al., 2022a). The present paper contributes to this debate by exploring whether the in–out effect is based on an internal standard of consonant order. To investigate this, we exploit the difference in judgmental formation in between-subject and within-subject designs. In this way, we can test different mechanisms that have been put forward to explain the in–out effect.

## Judgment formation in between- and within-subject designs

In a between-subjects design (with a single trial being the most extreme case), individuals are exposed to only one manifestation of the experimental conditions or a single stimulus (cf., Keren, 2014). In such designs, individuals have only the stimulus features and their accompanying processing dynamics to inform their judgment. These cues must deviate strongly enough from some internal standard participants maintain in their mind to affect judgments (Kahneman & Miller, 1986; Macedo & Cardoso, 2019; Mussweiler, 2003). Also, this standard itself must be strong enough to trigger signals when being violated (e.g., Bless & Burger, 2016; Gerten & Topolinski, 2019; Rumelhart & Ortony, 1977). In a within-subjects design, however, individuals are exposed to several manifestations of the experimental conditions and several stimuli (cf., Keren, 2014). In such designs, in addition to the basic features of each given stimulus individuals have the relative differences between the various stimuli to calibrate their judgments along the trials (cf., calibration in serial evaluative judgments, Fasold et al., 2012; Unkelbach & Memmert, 2014; Unkelbach et al., 2012).

This difference between within- and between-subjects designs can be used to explore the presence and strength of an internal standard. If a certain effect occurs in a one-shot between-subjects design, there must be a strong internal standard. For instance, the word LOVE should be preferred over the word HATE already in a one-trial between-subjects design. Each of these words triggers an affective response deviating from our usual neutral affective baseline, a strong experiential internal standard. To use a psychological manipulation closer to the in–out effect, the letter string CODROT should be preferred over the letter string OTCDRO (both being anagrams of DOCTOR, Topolinski et al., 2016) also in a between-subjects design because the latter starkly defies the strong internal standards we hold about word syllable structure, vowel-consonant alternation, and thus resulting pronounceability (cf., Laham et al., 2012; Newman et al., 2014; Song & Schwarz, 2009).

In contrast, there are stimulus(-related) features for which individuals hold no absolute strong standard but rather establish the standard according to the relative level of and the differences in manifestations between the stimuli they encounter. One example that has been researched in detail in recent years is the ease with which a stimulus can be processed (processing fluency, e.g., Reber et al., 2004, Topolinski & Strack, 2009a, 2009b). Manipulations of processing fluency usually employ within-subjects designs in which the effect unfolds over the course of trials, since individuals hold no strong internal standard of absolute fluency but rather develop this standard given the stimulus ecology they encounter (see Dechêne et al., 2009; Garcia-Marques et al., 2019; Hansen et al., 2008; Wänke & Hansen, 2015; but also see exceptions regarding the truth-effect and repetition reviewed by Garcia-Marquez et al. 2019).^1^

From these considerations, it follows that if a certain effect occurs in a one-trial between-subjects design, the target stimulus must deviate strongly enough from a pre-existing strong internal standard or scheme. Applied to the in–out effect, if consonantal articulation direction influences liking even in a one-trial between-subjects design, individuals need to hold an internal standard of how consonantal articulation places follow each other in words. Also, this standard must be strong enough to elicit affective responses when stimuli violate it. The qualification that the deviation of the stimulus from that internal standard must also be strong enough to be effective can be ignored since there cannot be a stronger deviation from a sequence than its complete reversal (inward vs. outward).

This logic of testing the in–out effect in a one-trial between-subjects design informs our theorizing on its underlying mechanisms since the different explanations of the in–out effect put forward differ regarding the strength of such an internal standard or scheme of consonant order. Let us consult each of these explanations and their respective predictions.

## The internal standard of consonant order in different theories of the in–out effect

First, it has to be emphasized that an internal standard or scheme of consonant order is not a noetic rule or conscious belief individuals are aware of (Ingendahl et al., 2022a,2022b,2022c,2022d). When being probed, participants never show insight into the in–out manipulation; when being debriefed, participants never mention consonant order as a cue they based their judgments on. It is rather a phonotactic (cf., e.g., Onishi et al., 2002) or mere motor constraint (MacDonald & Weiss, 2022) in our schemata of language representation and influences the encoding of verbal material on a very basic, fast, and automatic level. Also, the present work does not deal with the question of whether individuals become aware of the experimental manipulation in within-subjects designs or not and whether this may produce an effect (for a discussion, see Keren, 2014).

The eating-related embodied account (Godinho et al., 2019a,2019b; Maschmann et al., 2020; Topolinski & Boecker, 2016b; Topolinski et al., 2017) states that the oral motor sequences during subvocalization of consonants resemble eating (inward words) or expectoration (outward words) mouth movements that are positively or negatively associated. These overlearned, instinctive, phylogenetically primeval, and ontologically very early motor sequence representations should be a very strong internal standard. Also, they represent absolute rather than relative or graded representations: An inward motor trajectory is associated with appetitive food consumption movements, an outward trajectory is associated with aversive expectoration movements, and there is rarely a mixed or graded behavior (e.g., sucking one’s own sputum). Thus, this account would predict in–out effects for one-trial between-subjects designs, it should be only a matter of statistical power.

The processing fluency and language frequency account (Bakhtiari et al., 2016; Godinho & Garrido, 2021; Ingendahl et al., 2021, 2022a,2022b,2022c,2022d; Körner et al., 2019) states that inward relative to outward words are easier to articulate (cf., Silva et al., 2017; Song & Schwarz, 2009; Topolinski & Strack, 2010), because their consonantal sequence structure imitates real words in natural language more closely. Indeed, in corpus analyses it was found that inward consonant sequences are slightly more frequent than outward sequences in real language (Bakhtiari et al., 2016). However, since real words rarely feature pure inward or outward sequences of consonants, but rather language shows a slight bias toward inward sequences across many words at best (Bakhtiari et al., 2016), this internal language-based standard should be rather weak and subtle. Thus, a fluency/frequency account would predict the absence of an in–out effect in one-trial between-subjects designs. Rather, it would predict that the effect would unfold over the course of several trials involving both inward and outward words, which makes the deviations from that subtle internal standard salient eventually.

A more specialized version of a fluency account focusing directly on motor constraints is easy first in language acquisition (Ingendahl et al., 2022a; MacDonald & Weiss, 2022; Topolinski et al., 2022), stating that from ontogenetically early speech production on, front consonants are produced more often and are thus motorically easier to pronounce than back consonants, and individuals prefer processing easy before harder motor components. Since this well-established labial-coronal effect emerges very early in language acquisition and seems to be linguistically universal (for a recent review, see Aoyama & Davis, 2016), it should maintain a very strong internal standard during word processing. Thus, this account would predict in–out effects in one-trial between-subjects designs.

Finally, the letter-position account (Ingendahl & Vogel, 2022b; Maschmann et al., 2020) states that front consonants occur more often in the beginning than at the end of real words in natural language and readers pay more attention to word beginnings than the rest of a word (for discussions to what degree reader focus on word beginnings and endings, respectively, see Ingendahl & Vogel, 2022b; Maschmann et al., 2020). Similar to the fluency/frequency account, this would yield a rather subtle internal standard. Consequently, this account would predict in–out effects only in within-subject designs.

## Present research

The aim of the present work is to shed light on the underlying mechanism of the in–out effect. Gauging in–out effects in between-subjects designs and comparing their effect size to within-subject designs yields highly informative results that do not ultimately decide between the different explanations of the in–out effect, but still provide support and counterevidence for the respective theories (cf., e.g., Fawcett, 2013, for a similar consideration to explore the production effect). While the eating-related embodiment and easy-first account would predict between-subjects in–out effects, the fluency/frequency and letter-position accounts would not.

In Experiments 1a–e, we realized between-subjects replications of earlier within-subjects designs of the in–out effect in three different languages. In Experiment 2, we tested the in–out effect for different numbers of items in a within-subjects design and gauged its development over the course of the initial trials.

## A priori power-analyses, transparency, and openness

Assuming a small-to-moderate effect size of *d* = 0.3 (requiring *N* = 352 to detect a possible in–out effect with a power of 0.80), Experiment 1a was conducted with the aim to collect at least 350 participants but as many participants as possible given a certain logistic time frame (availability of the laboratory). Eventually, *N* = 437 participants were collected yielding *d* = 0.28 (*N*_required*|*0.80_ _power_ = 404). For the online setup in Experiment 1b a pilot study (*N* = 49, 29 male, 18 female, 2 diverse; mean age 10, SD = 10) was conducted with the same method as the eventual experiment. This yielded inward (*n* = 21) = 4.86 (SD = 2.29), outward (*n* = 28) = 4.14 (SD = 1.78), *d* = 0.36, 95% CI [− 0.22 to 0.92]. This extrapolates to *N*_required|0.80 power_ = 246, *N*_required|0.95 power_ = 404. Thus, we aimed to assure a power of 0.95 and collected at least *N* = 400 in Experiments 1b–e.

For the within-between interaction in Experiment 2, we powered (0.80) for a small to medium effect (*f* = 0.15), leading to a minimal sample size of *N* = 140 (28 per condition). Due to uncertainty about the actual effect size, we oversampled up to 250 participants.

We report how we determined our sample size, all data exclusions (if any), all manipulations, and all measures in the studies—and we report all studies we conducted for this setup. Data were analyzed using SPSS, Bayesian tests were conducted in JASP 0.16.1 with default settings.

## Experiments 1a–e

### One-trial between-subjects designs in German, English, and Portuguese

First, we explored the possibility of an in–out effect involving only one trial per participant. The only previous publication we are aware of that employed a between-subjects design of the in–out effect was Rossi et al. (2017). However, those authors used a stimulus pool of only 16 in/out letter strings and sampled 3 of them for each participant in the in- and the out-group, respectively.

We used stimulus material that has yielded reliable in–out effects in earlier within-subjects designs adapted to German (Experiments 1a, b), English (Experiment 1c), and Portuguese (Experiment 1d) phonation, respectively. Since the in–out effect is assumed to be a universal phenomenon (Topolinski et al., 2014) and has indeed been replicated in various languages, even outside the Indo-European language family and in non-WEIRD samples (Godinho et al., 2019a,2019b; Motoki & Pathak, 2022), the present test involved three different languages to attest its cross-language generalizability.

In addition, we report within-subjects replications with the respective materials used in Experiments 1a–c. To rule out that in a one-shot between-subjects design a missing in–out effect is due to the circumstance that participants were not prepared, thus inattentive, or not familiarized with the liking rating for that one stimulus, we implemented a warming-up phase with some irrelevant non-verbal filler items in Experiments 1b–d.

Moreover, in Experiment 1e we employed a manipulation of pronounceability of letter strings (cf., Laham et al., 2012; Newman et al., 2014; Song & Schwarz, 2009) in addition to in/out strings. An independent group of participants was presented with one either easy-to-pronounce letter string (e.g., BATREK) or one hard-to-pronounce letter string (e.g., EAKRTB; Silva et al., 2017; Topolinski et al., 2016; Zürn & Topolinski, 2017). Given our assumption that pronounceability has a relatively strong internal standard (see introduction), a manipulation of pronounceability should also work in a between-subjects design (note that this manipulation is usually employed within-subjects).

Experiment 1a was a laboratory study from 2017 with German participants whose demographics and raw data were lost in a rushed evacuation of the laboratory during the Covid outbreak in the spring of 2020. Still, we report this data set to avoid file drawer issues.

## Methods

### Samples

In Experiment 1a, *N* = 437 (demographics were lost in the laboratory evacuation during the Covid outbreak in spring 2020) individuals recruited from the campus of the University of Cologne, Germany, took part in an onsite laboratory experiment for candy as a reward. In Experiment 1b, *N* = 397 (190 female, 196 male, 11 diverse; mean age 28, SD = 8) German native speakers were recruited via Prolific Academic (https://www.prolific.co) in an online setting and participated for a reward of ~ 0.35£. In Experiment 1c, *N* = 549 (316 female, 228 male, 2 diverse, 3 prefer not to say; mean age 43, SD = 14) English native speakers were recruited via Prolific Academic for an online survey for an 8€/h reward. In Experiment 1d, *N* = 490 (237 female, 248 male, 5 diverse; mean age 26, SD = 8) Portuguese native speakers were recruited via Prolific Academic for an online survey for 0.23£ reward. In Experiment 1e *N* = 1100 (651 female, 426 male, 21 diverse, 2 prefer not to say; mean age 38, SD = 13) English native speakers were recruited via Prolific Academic for an online survey for an 8€/h reward.

### Materials

#### In–out stimuli

In Experiments 1a and 1b, we used the stimulus pool adjusted to German phonation provided in Topolinski and Boecker (2016a; Experiment 1), which yielded reliable effects in within-subjects implementations (e.g., Silva & Topolinski, 2018). These stimuli consist of two-syllable vocal-consonant-vocal-consonant (VCVC) letter strings each featuring a front (B, M, P) and a back consonant (G, K, R) either in the inward direction (front-back) or the outward direction (back-front). All possible combinations of the respective three front and back consonants were realized and all possible filler vowel combinations (vowels A, E, I, O, U).^2^ The resulting stimuli were *N* = 68 inward and *N* = 72 outward stimuli. In Experiment 1c, we used the stimulus pool adjusted to English phonation provided in Topolinski et al., (2014, Experiment 6) that used front (B, F, M, P), middle (D, L, N, S, T), and back (K) consonants and random vowels to construe VCVCVC, VCVCVCV, CVCVC, and CVCVCV letter strings. The resulting pool contained *N* = 125 inward and *N* = 157 outward stimuli. In Experiment 1d, we used the stimulus pool adjusted to Portuguese phonation provided by Godinho and Garrido (2016) involving front (P, B, F, V), middle (T, N, D), and back (C, G) consonants and random vowels in CVCVCV and VCVCVC structures. The pool contained *N* = 138 items of each inward and outward stimuli, respectively. In all of these pools, meaningful words were discarded, of course.

#### Filler stimuli for warming-up phase

In Experiments 1b-d, we used a Chinese ideograph (taken from Payne et al., 2005), a foto of a woman’s face with a neutral expression (taken from Goeleven et al., 2008), a polygon (source unknown), and a dot pattern (taken from Hansen & Topolinski, 2011).

#### Pronounceability stimuli

In Experiment 1e, we used the stimulus pool of easy vs. hard-to-pronounce letter strings published in Topolinski et al. (2016). These consisted of anagrams of real German words (e.g., BETRAG, amount) that were rendered to be either easy (BATREG) or hard to pronounce (EAKRTB). The stimuli ranged from 6 to 11 letters and amounted to 50 per condition. These stimuli have shown reliable and strong effects in within-subjects designs on anagram solvability ratings (Topolinski et al., 2016), the trustworthiness of eBay usernames (Silva et al., 2017), and money transfer in the trust game (Zürn & Topolinski, 2017).

#### Procedure

In all experiments, participants were informed that they would be presented stimuli they should evaluate for future research on a Likert scale ranging from (1^3^) I do not like it at all to (10) I like it a lot. In the onsite laboratory Experiment 1a, they were instructed that they would be shown a single nonsense word to read and evaluate. In the online Experiments 1b-1e, they were instructed that they would be shown several stimuli (the nature of those stimuli, e.g., being either visual or verbal, was not specified). As a warming-up phase before the crucial evaluation of the in/outward target word, they evaluated the abovementioned visual filler stimuli in the fixed order they are mentioned in the materials section above (these ratings were not recorded). We implemented this warming-phase to make sure participants paid full attention to the crucial letter string trial in this online setup.

In the crucial target trial in each experiment, one random stimulus from the inward and outward pool was randomly selected per participant. In addition, in Experiment 1e a group of participants rated their liking of an easy or hard-to-pronounce letter string. The tasks took less than 5 min.

Materials and data are available online at https://osf.io/a5n6t/?view_only=7306671bef694d4fa5d69197cf698855. The preregistrations for Experiments 1b-e can be found at https://aspredicted.org/ZLL_YXC (1b), https://aspredicted.org/PTK_2DN (1c), https://aspredicted.org/N85_6RN (1e). We have complied with APA ethical standards in the treatment of our sample.

## Results

As can be seen in Table 1, the evidence for a preference of inward over outward letter strings was mixed across the different experiments. A joint ANOVA including all experiments found no in–out effect, *F*(1, 2412) = 0.13, *p* = 0.715, *η*_p_^2^ < 0.01, a main effect of experiment, *F*(4, 2412) = 22.93, *p* < 0.001, *η*_p_^2^ = 0.04, and no interaction, *F*(4, 2412) = 2.01, *p* = 0.091, *η*_p_^2^ < 0.01. The main effect of experiment was constituted by the fact that the liking ratings were overall higher in the onsite laboratory Experiment 1a than in the other experiments. In essence, there was no difference in liking between inward (*M* = 3.97, SD = 1.97) and outward letter strings (*M* = 4.03, SD = 2.00), *t*(2420) = 0.72, *p* = 0.474, *d* = − 0.03, 95% CI (*d*) (− 0.11, 0.05), BF_10_ = 0.059.

**Table 1:** Statistics for Experiments 1a–e

Experiment	Articulation Direction		*df*	*t*	*p*	*d*	95% CI (*d*)	BF_10_
	Inward	Outward						
1a	4.79 (1.30) *N* = 217	4.44 (1.23) *N* = 220	435	2.90	0.004	0.28	0.09, 0.47	5.98
1b	3.91 (1.83) *N* = 200	3.88 (1.91) *N* = 197	395	0.12	0.908	0.12	− 0.19, 0.21	0.11
1c	3.89 (2.26) *N* = 276	4.23 (2.22) *N* = 273	547	− 1.78	0.076	− 0.15	− 0.32, 0.02	0.44
1d	3.33 (1.80) *N* = 254	3.47 (2.02) *N* = 236	488	− 0.79	0.431	− 0.07	− 0.25, 0.11	0.14
1e	4.03 (2.13) *N* = 274	4.08 (2.20) *N* = 275	547	− 0.02	0.814	− 0.02	− 0.19, 0.15	0.10

All means were based on a 10-point rating scale, except for Experiment 1c and 1e with an 11-point scale. Standard deviations appear in parentheses

*N*  number of participants, *df*  degrees of freedom, *d*   Cohen’s *d*, *BF*_*10*_  Bayes Factor for a Bayesian *t* test in JASP. The values for Experiment 1e only pertain to the two groups who received in/out stimuli in that experiment. The results for the groups receiving (dys)fluent stimuli are reported in the text

In contrast, in Experiment 1e, regarding the additional groups being presented with one easy- or one hard-to-pronounce letter string, participants marginally preferred easy (*M* = 3.54, SD = 2.14) over hard letter strings (*M* = 3.20, SD = 2.30), *t*(549) = 1.86, *p* = 0.063, *d* = 0.16, 95% CI (*d*) (− 0.01, 0.33). Also, participants overall preferred inward/outward (*M* = 4.05, SD = 2.17) over easy/hard letter strings (*M* = 3.37, SD = 2.14), *F*(1, 1096) = 27.95, *p* < 0.001, *η*_p_^2^ = 0.03.

To attest that the stimuli used in the present between-subjects designs are effective, we report a within-subjects online study with the German stimulus pool from Experiments 1a–b on German native speakers (*N* = 16). This yet unpublished data set was a pilot study for the baseline of another, conceptually unrelated project (judgmental correction of the in–out effect, Topolinski & Löffler, 2022), randomly sampling 30 inward and 30 outwards words from the Topolinski and Boecker (2016a, Experiment 1) stimulus pool per participant. In this data set, participants liked inward letter strings (*M* = 4.64, SE = 0.27) more than outward letter strings (*M* = 4.30, SE = 0.24), *t*(15) = 3.09, *p* = 0.008, *d*_*z*_ = 0.77, 95% CI (*d*_z_) (0.20–1.32).

Moreover, we report a preregistered (https://osf.io/r72ed/?view_only=3c3c2dce81814b1794fa1691ae71b4ed) unpublished within-subjects online study with the English stimulus pool from Experiment 1c (from Topolinski et al., 2014, Experiment 6) that sampled 12 inward and 12 outward words per participant. In this data set, participants liked inward letter strings (*M* = 4.03, SE = 0.14) more than outward letter strings (*M* = 3.90, SE = 0.13), *t*(252) = 2.97, *p* = 0.003, *d*_*z*_ = 0.19, 95% CI (*d*_z_) (0.06–0.31).

## Discussion

We went to great lengths to document an in–out effect that involves only one trial, testing 2422 participants in three languages. We did not establish such evidence, although the stimuli used in the present experiments were carefully crafted according to the respective phonation rules in German, English, and Portuguese and had produced reliable in–out effects in within-subjects designs in previous publications, and proved reliable in additional within-subjects replications reported here (for Experiments 1a–c).

Furthermore, according to our knowledge being the first to implement a pronounceability manipulation in a one-shot between-subjects design, we did find a marginally significant effect (Experiment 1e). We argue that this was the case because pronounceability bears an internal standard stronger than consonant order, and participants do not need several stimuli to calibrate their liking but use their internal standard of word structure in the single trial right away. Of course, given the very low item reliability of this single measurement, the effect was very small. Also, the items used in that experiment derive from German words, and thus the psycholinguistic properties of those stimuli aside from pronounceability (e.g., letter frequencies) were unfamiliar to the English sample. This is reflected in the fact that the participants in Experiment 1e preferred in/out letter strings generally over (dis)fluent letter strings (the former being specially crafted for English phonation). In turn, the very fact that there was a solid between-subjects difference in the liking of in/out vs. (dis)fluent letter strings supports the idea that such effects are possible in one-shot between-subjects designs.

The only two differences between Experiment 1a and the other experiments, namely being an onsite laboratory experiment and doing without the warming-up evaluation phase, can hardly explain why Experiment 1a found an in–out effect and the others (online and with a warming-up phase) did not. First, the additional two studies attesting a within-subjects in–out effect were also online studies; and second, it is implausible that the presentation of four neutral stimuli in advance should block the in–out effect.

## Experiment 2

### Different numbers of items in a within-subjects design

We employed a within-subjects manipulation of the in–out effect with varying numbers of items per in/out category to explore (a) how the in–out effect depends on the number of items, and (b) how the in–out effect unfolds over time. In past publications, this number of items (sampled from usually much larger stimulus pools) ranged from 10 (Topolinski et al., 2014, first experiments) to up to 60 items per category per participant (Topolinski & Boecker, 2016a, 2016b, Experiment 2b). Here, we manipulated between-subjects whether 1, 5, 10, 30, or 50 items per in/out category were presented in a within-subjects design. In addition, independent of the between-subjects condition of how many items a participant received overall, we analyzed only the first 1, 2, 3, up to 10 trials in the whole data set to see how the in–out effect unfolds over time (for a similar approach focusing on the initial and last trials in a truth effect experiment, see Garcia-Marquez et al., 2019).

## Methods

### Sample

*N* = 253 (demographics were lost in the laboratory evacuation during the Covid outbreak in spring 2020) individuals were recruited from a campus of the University of Cologne, Germany, and participated in this onsite laboratory experiment for €3 as a reward (the task was always bundled with other, conceptually irrelevant tasks, see procedure section).

### Materials

The 2-syllables stimulus pool for German phonation from Topolinski and Boecker (2016a; Experiment 1) was used (as in Experiments 1a, b).

### Design

We employed a 2 (articulation direction: inward, outward; within-subjects) X 5 (number of items per within-condition: 1 item, 5 items, 10 items, 30 items, 50 items; between-subjects) design.

### Procedure

Since the different between-subjects conditions differed in length, the present tasks were always bundled with other, conceptually irrelevant tasks (Gerten & Topolinski, 2021; Gerten et al., 2022; Zürn et al., 2021) in variable length and flexible combination to always end up with an experimental session of approx. 15–20 min. This was done to rule out that possible differences in the present conditions were due to differing participant rewards and thus motivation. In the present task, participants were informed that they would be presented with nonsense words and should report their spontaneous liking for each stimulus typing in a number ranging from (0) I do not like it at all to (10) I like it a lot. Then, participants received either 1, 5, 10, 30, or 50 inward and outward words, respectively, in random order re-randomized anew for each participant to render their liking ratings. The tasks took 1 to 7 min.

Data are available online at https://osf.io/a5n6t/?view_only=7306671bef694d4fa5d69197cf698855. We have complied with APA ethical standards in the treatment of our sample.

## Results and discussion

Mistyped responses (14 of 9670, 0.1%) were discarded (the experimental driving software DirectRT allowed not only to type in numbers but also letters). Two participants in the 1-item per category condition typed in invalid responses in both trials and were discarded. One participant from the 1-item per category condition typed in an invalid response in one trial and was thus discarded from the analyses in Table 2, but was included in the later exploratory analyses in Fig. 3 (see below).

**Table 2:** Statistics for the experimental between-subjects conditions in Experiment 2

Number of items per in–out condition	Articulation Direction		*df*	*t*	*p*	*d*_*z*_	95% CI (*d*_z_)	BF_10_
	Inward	Outward						
1 item (*N* = 47)	4.28 (0.35)	3.72 (0.28)	46	1.48	0.145	0.22	-0.07, 0.50	0.44
5 items (*N* = 49)	4.63 (0.24)	4.20 (0.22)	48	2.39	0.021	0.34	0.05, 0.63	2.01
10 items (*N* = 54)	5.18 (0.19)	4.60 (0.20)	53	4.61	< 0.001	0.63	0.33, 0.92	770.34
30 items (*N* = 50)	4.60 (0.17)	4.30 (0.18)	49	5.11	< 0.001	0.72	0.41, 1.03	> 1000
50 items (*N* = 50)	4.56 (0.20)	4.15 (0.19)	49	4.76	< 0.001	0.67	0.36, 0.96	> 1000

All means were based on a 10-point rating scale. Standard errors appear in parentheses

*N*   number of participants, *df*  degrees of freedom, *d*_*z*_  Cohen’s effect size for within-subjects *t* tests, *BF*_*10*_  Bayes Factor for a Bayesian *t* test in JASP

First, we tested whether the number of items presented in each between-subjects condition affects the in–out effect, which should result in an interaction of articulation direction and item number. A 2 (articulation direction: inward, outward; within-subjects) X 5 (number of items per within-condition: 1 item, 5 items, 10 items, 30 items, 50 items; between-subjects) ANOVA over the aggregated liking ratings found a main effect of articulation direction, *F*(1, 245) = 28.07, *p* < 0.001, *η*_p_^2^ = 0.10; a conceptually irrelevant main effect for number of items, *F*(4, 248) = 2.47, *p* = 0.045; and no interaction, *F*(4, 245) = 0.36, *p* = 0.834. Across groups, inward letter strings (*M* = 4.66, SE = 0.11) were liked more than outward letter strings (*M* = 4.20, SE = 0.10), *t*(249) = 5.34, *p* < 0.001, *d*_*z*_ = 0.34, 95% CI (*d*_z_) (0.21–0.47).

Despite the absent interaction, we plotted the respective descriptive and *t* tests per between-subjects condition in Table 2 for illustrative reasons. When only 1 item per category was presented, the in–out effect emerged with an already non-neglectable but insignificant effect size of *d*_*z*_ = 0.22, increased in effect size approaching significance (mind Bonferroni-correction for multiple testings) when 5 items per category were presented and reached a solid plateau of highly significant effects for 10 to 50 items per category. This pattern is not surprising for mere statistical but also for psychological reasons: Of course, the more items are sampled, the higher the reliability of the measurement and thus the effect size (e.g., Bellemare et al., 2014; Erlebacher, 1977; Judd et al., 2012; Lakens, 2013). However, note that in the present data there is no pattern of an ever increasing effect size with increasing number of items: with 10 items per category the effect size reaches a plateau.

**Fig. 1:**
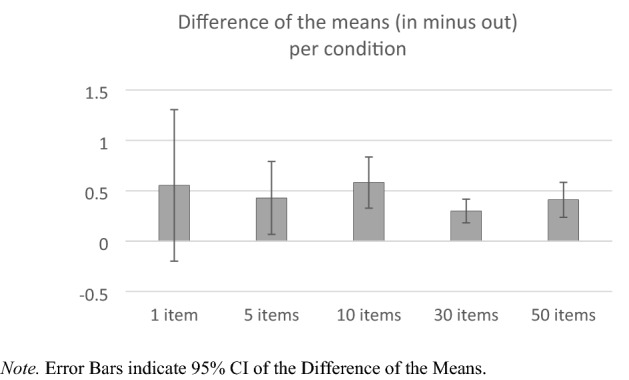
The Difference of the Means between Inward and Outward Trials per Experimental Between-Subjects Condition in Experiment 2. Note. Error Bars indicate 95% CI of the Difference of the Means

The more interesting aspect for the present exploration is the immediate onset of the in–out effect already in the 1-item per category condition. Figure 1 displays the differences of the means (inward minus outward) per number-of-items condition. These measures illustrate how much the ratings deflect contingent on in/out items and thus map the sensitivity (in a psychological, not statistical meaning) to the in/out manipulation. Independent from the decreasing error margins along with the conditions (which is due to the increasing measurement reliability mentioned above), the raw mean of the difference between inward and outward trials is already relatively high in the 1-item condition and does not differ between the groups (thus the missing interaction in the ANOVA).

### Analyses on a trial-level

**Fig. 2:**
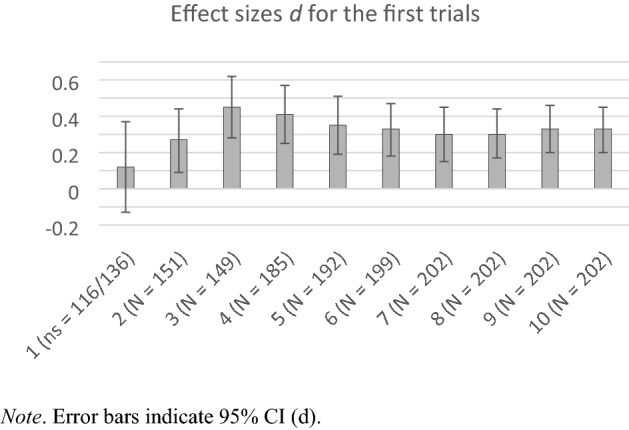
The Effect sizes d of the In–Out Effect for the Respective First, First 2, First 3, up to First 10 trials in Experiment 2 Across Between-Subjects Groups. Note. Error bars indicate 95% CI (*d*)

Next, we analyzed how the in–out effect unfolds over the course of trials, irrespective of the between-subjects condition of how many items a participant received overall. For instance, for a participant both in the 1-item and 50-items per category group, the very first trial of the task works psychologically like a one-trial between-subjects design (Experiments 1a-e). Also, this analysis allows for including much more participants than the above analyses that split the sample into groups. The resulting in–out effect sizes are shown in Fig. 2.^4^

Perfectly mirroring the pattern from the main analysis (that, however, involved fewer participants) and the evidence from Experiments 1a-e, the first trial does not show a noteworthy in–out effect (*d* = 0.12). Then, already with the second trial, the effect size doubled (*d* = 0.27) and was, in this analysis, significant, *p* = 0.003. This evidence is conceptually similar to the 1-item per category (i.e., 2 items altogether) group in Table 2, which had a similar effect size but was not significant due to the smaller sample size. Finally, the in–out effect soon settles on highly significant (all *p*s < 0.001) effects well above *d* = 0.30 within the first ten trials. This evidence is conceptually similar to the 5-item per category (i.e., 10 items altogether) group in Table 2 that had a similar effect size but only marginal significance. Again, this pattern is conflated with increasing reliability due to the increasing number of items involved, however, there is no monotonous increase.

**Fig. 3:**
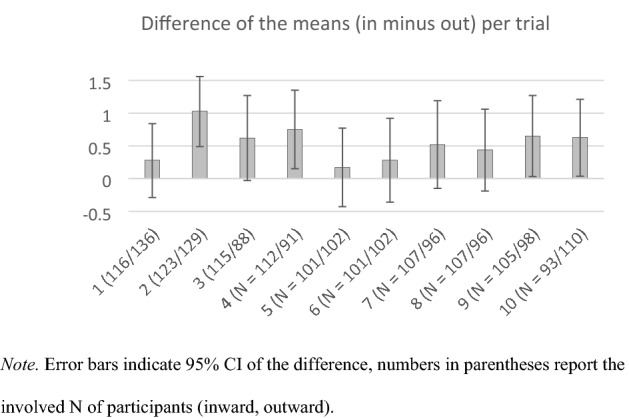
The Difference of the Means (inward minus outward) Plotted Separately for each of the Ten First Trials in Experiment 2. Note. Error bars indicate 95% CI of the difference, numbers in parentheses report the involved *N* of participants (inward, outward)

To map the sensitivity to the in–out manipulation per trial independent of item number/measurement reliability, Fig. 3 displays the differences of the means (inward minus outward) separately for each of the first ten trials (which of course can only be done via between-subjects tests). Here again, the liking ratings already show a sensitivity to the in–out manipulation starting with the second trial.

### The trick of the second trial

**Fig. 4:**
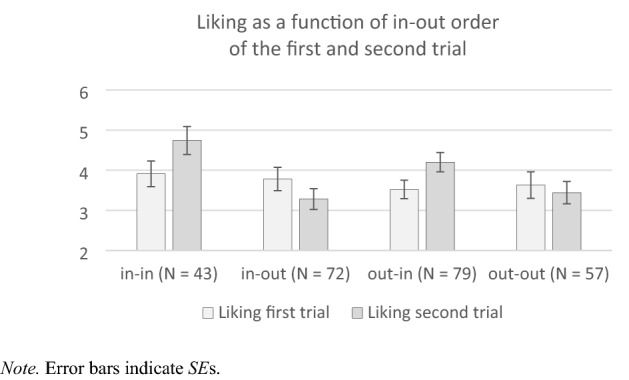
Liking Ratings as a Function of which Item Category (inward or outward) a Participant Received in the First and Second trial. Note. Error bars indicate SEs

For exploratory reasons, we analyzed the liking ratings across all participants for the first and the second trial, respectively, contingent on which in–out category they were presented in the given trial. Since in–out was randomly sampled, there were four groups: participants who received first an inward and then again an inward item, first an inward and then an outward item, first an outward and then an inward item, and first an outward and then again an outward item. The means are displayed in Fig. 4.

The interaction consonantal order (inward vs. outward) X trial number (first trial vs. second trial) was significant, *F*(3, 247) = 4.88, *p* = 0.003, *η*_p_^2^ = 0.06. The likings ratings did not differ between the groups in the first trial (white bars in Fig. 4), *F*(3, 248) = 0.36, *p* = 0.784, but in the second trial (grey bars in Fig. 4), *F*(3, 248) = 5.45, *p* = 0.001, *η*_p_^2^ = 0.06. For participants receiving first an inward and then again an inward item, liking ratings increased from the first to the second trial, *t*(42) = 2.62, *p* = 0.012, *d*_*z*_ = 0.40, 95% CI (*d*_z_) (0.09, 0.80). Thus, although inward order did not affect the first liking rating, it added positive valence to the liking in the second trial. For participants receiving first an inward and then an outward item, liking ratings dropped only descriptively across trials, *t*(71) = 1.65, *p* = 0.100, *d*_*z*_ = 0.20, *95% *CI (*d*_z_) (− 0.04, 0.43). Thus, a contrastive detrimental effect of outward on liking was not observed. For participants receiving first an outward and then an inward item, liking ratings increased across trials (despite Bonferroni correction for multiple testing), *t*(78) = 2.65, *p* = 0.010, *d*_*z*_ = 0.30, *95% *CI (*d*_z_) (0.07, 0.52). Thus, a contrastive beneficial effect of inward on liking was observed. For participants receiving first an outward and then again an outward item, liking ratings did not differ across trials, *t*(56) = 0.65, *p* = 0.521, *d*_*z*_ = 0.09, 95% CI (*d*_z_) (− 0.18, 0.35). Thus, outward consonant order did not add negative valence to the liking in the second trial. In sum, effects of the in–out manipulation were absent in the first, but emerged already in the second trial, particularly a beneficial additive and contrastive impact of inward on likings, while outward had a lesser pronounced detrimental effect on preferences.

## General discussion

We explored the strength of the internal standard or schema individuals hold about consonantal order in random letter strings by comparing the in–out effect sizes in between- and within-subjects designs. If individuals carry a strong internal standard of inward and outward consonantal order and the respective affective connotations, as the eating-related embodiment (Topolinski et al., 2014) and the easy-first (MacDonald & Weiss, 2022) account suggest, then the in–out effect would already occur in a one-trial between-subjects design, similar to other effects with strong internal comparison standards. Testing over 2400 participants in three different languages, we did not find such an effect (Experiments 1a-e). This null-finding is unlikely due to lacking power since it was corroborated by Bayes analyses that attested strong evidence against H1, BF_10_ = 0.059. Also, as a whole, Experiments 1a-e had a post-doc power of (1−*β*) = 0.998 to detect a small effect size *d* = 0.2, and still (1−*β*) = 0.958 to detect an effect size of *d* = 0.15. In contrast, a manipulation of pronounceability of letter strings, which assumably involves a stronger internal standard of word structure, produced a marginal between-subjects effect (Experiment 1e).

In Experiment 2, employing within-subject replications of the in–out effect with varying item numbers, we again found no in–out effect for the first trial (which equals a one-trial between-subjects instantiation psychologically). However, already in the second trial, the in–out effect began to emerge with highly significant effects in the analyses involving larger samples. Particularly inward items increased liking, but outward items did not significantly decrease liking. Across the further course of the liking task, the in–out effect very soon reached a plateau of solid significant effect sizes within the first few trials (see Fig. 2).

This pattern resembles the unfolding of any fluency effect over the course of an experiment, where no absolute standards to compare the stimulus with are available, but participants develop an internal standard given the relative differences of the stimuli they are being presented with (cf., Dechêne et al., 2009; Garcia-Marques et al., 2019; Hansen et al., 2008; Unkelbach et al., 2012; Wänke & Hansen, 2015). Most importantly, this pattern would be predicted by both the fluency/frequency (e.g., Bakhtiari et al., 2016; Godinho & Garrido, 2021; Ingendahl et al., 2021, 2022a, 2022b, 2022c, 2022d; Körner et al., 2019) and the letter-position (Ingendahl & Vogel, 2022b; Maschmann et al., 2020) accounts of the in–out effect.

Yet, the present analyses also showed that the in–out effect emerges surprisingly fast, already in the second trial. Particularly the finding that a second inward item presented after an initial inward item increased likings points to the direction of a possible absolute judgmental standard instead of relative judgmental calibration. This should be scrutinized in the future with larger sample sizes.

As a limitation, it is also necessary to consider the role of measurement reliability in the present findings. It is almost trivial that the in–out effect is smaller for a single measurement than for multiple trials. Yet, a mere reliability explanation would predict a monotonously asymptotic increase of the in–out effect with an increasing number of trials (up to the “true” effect size). This is not what we observe in Experiment 2 (see Fig. 2), where the in–out effect is strongest already for the three first trials and even slightly decreases afterwards. Also, the results of Experiment 1e show that even a single measure in a pure between-design can detect systematic preferences between word stimuli—namely between the in/out and (dis)fluent letter strings.

As another limitation, the present study is not a decisive test of the competing theories about the driving mechanisms of the in–out effect, but it provides further evidence to the ongoing conceptual debate (Ingendahl et al., 2022b). It renders an eating-related embodiment (see Maschmann et al., 2020) and an easy-first account (see Topolinski et al., 2022) even more unlikely and puts the in–out effect into the conceptual vicinity of usual fluency effects and their dependence on several sequential stimulus presentations (e.g., Bakhtiari et al., 2016; Dechêne et al., 2009).

Concluding, a fluency account of the in–out effect seems a promising explanation. However, recent research has shown that central predictions of a fluency account, a part of the presence of an internal standard, could not be supported experimentally (Ingendahl et al., 2022a,2022b,2022c,2022d). For instance, in mediation analyses, the impact of consonant order on liking is only partially mediated by simultaneously assessed subjective fluency (Bakhtiari et al., 2016), and the in–out effect is not modulated by experimental inductions of fluency using training (Ingendahl et al., 2021). Thus, further research is needed to determine the actual driving force behind the in–out effect.

### Implications for other psycholinguistic effects and marketing

Beyond the examination of the in–out effect, the present logic of comparing between-subjects and within-subjects designs to gauge the role of an internal judgmental standard in a certain effect can be useful in theory development for other phenomena as well. Generally, this logic can be used to test whether a certain effect is based on fluency or not (cf., Dechêne et al., 2009; Garcia-Marques et al., 2019; Hansen et al., 2008; Wänke & Hansen, 2015). Moreover, this logic can be used to test the involvement of an internal standard in any phenomenon.

As one example, regarding sound symbolism, for instance, consider the well-known bouba/kiki effect (Köhler, 1970), where a spiky visual form is associated with a sharp-edged phonetic name sound and a round form with a round sound (for recent examinations, see, e.g., Barton & Halberstadt, 2018; Cuskley et al., 2017). Usually, this effect is tested in a one-shot between-subjects design (for a recent large-scale replication across languages using such a design, see Ćwiek et al., 2022). In contrast, the sound symbolism phenomenon of size-pitch association (small objects/high front vowels, large objects/low back vowels; Sapir, 1929) is usually tested in within-subjects designs (e.g., Klink, 2000). This might imply that the bouba/kiki-effect is driven by a stronger internal judgmental standard than usual sound symbolism effects, which should be tested in future studies.

The same applies to other psycholinguistics effects that are usually tested in within-subjects designs, such as vowel-affect (Rummer et al., 2014), voicedness-gender (Slepian & Galinsky, 2016), or sound-taste associations (Motoki et al., 2020). Particularly for their feasibility as a marketing technique (Topolinski, 2017a, 2017b), effects involving an internal standard evoking an effect already in a one-shot test are more promising as marketing tool as they are likely to elicit attitudes immediately during a single exposure to a single brand name.

## Data Availability

All data and materials are available at https://osf.io/a5n6t/?view_only=7306671bef694d4fa5d69197cf698855.

## Footnotes

1 Note that the previous example of pronounceability is usually conceptualized as an instantiation of processing fluency (e.g., Song & Schwarz, 2009). However, we argue that the internal standard of vowel-consonant alternation (resulting in pronounceability in human language) is much stronger than other stimulus features being subsumed under fluency, such as figure-ground contrast or semantic fluency of statements.

2 Generally, the in–out effect only concerns consonants, not vowels. Experimentally manipulating the order of front and back vowels in addition to consonant order, Topolinski & Boecker (2016a) found no vowel in–out effect, while Motoki & Pathak (2022) did.

3 In experiments 1c and 1e, the scale ranged from 0 to 10.

4 Note that the resulting participant sample sizes for particularly the first 2 and 3 trials are reduced since inward and outward words were presented in random order; and for the between-subjects conditions involving 5–50 trials it was possible that a participant received stimuli from only one category and was thus discarded from the analyses. Furthermore, from 3 trials onwards the between-subject group with only 1 item per category (i.e., 2 items overall) dropped from the analysis.
